# VEGFR1-tyrosine kinase signaling in pulmonary fibrosis

**DOI:** 10.1186/s41232-021-00166-7

**Published:** 2021-06-03

**Authors:** Hideki Amano, Yoshio Matsui, Ko Hatanaka, Kanako Hosono, Yoshiya Ito

**Affiliations:** 1 Department of Pharmacology, Kitasato University School of Medicine, 1-15-1 Kitasato, Sagamihara, Kanagawa, 252-0373 Japan; 2 Department of Thoracic Surgery, Kitasato University School of Medicine, Kanagawa, Japan

**Keywords:** Pulmonary fibrosis, VEGFR1-TK, VEGFR1^+^ cells, SDF-1, CXCR4

## Abstract

Vascular endothelial growth factor (VEGF) is not only an important factor for angiogenesis but also lung development and homeostasis. VEGF-A binds three tyrosine kinase (TK) receptors VEGFR1–3. Idiopathic pulmonary fibrosis (IPF) is one of the poor prognoses of lung diseases. The relationship of VEGF and IPF remains to be clarified. Treatment with nintedanib used for the treatment of IPF reduced fibroblast proliferation, inhibited TK receptors, platelet-derived growth factor receptor (PDGFR), fibroblast growth factor receptor (FGFR), and VEGFR. Because the effect of that treatment is still not satisfactory, the emergence of new therapeutic agents is needed. This review describes the enhancement of pulmonary fibrosis by VEGFR1-TK signal and suggests that the blocking of the VEGFR1-TK signal may be useful for the treatment of pulmonary fibrosis.

## Introduction

Idiopathic pulmonary fibrosis (IPF) is characterized by chronic, progressive, cellular proliferation, and interstitial inflammation fibrosis. IPF is a lethal lung disease with poor prognosis and occurs in adults but the exact mechanism of this disease is not fully understood [1]. During process of fibrosis, fibroblast proliferation amplifies extracellular matrix deposition and increases angiogenesis by the accumulation of macrophages [2]. Macrophages regulate tissue regeneration following injury [3]. They can worsen tissue injury by producing reactive oxygen species and other toxic mediators that disrupt cell metabolism, induce apoptosis, and exacerbate ischemic injury.

Angiogenesis, the phenomenon of growth of new capillary blood vessels in the body, is an important natural process for wound healing and regeneration of new tissue [4]. Abnormal blood vessel growth is responsible for the development of deadly and debilitating conditions like cancer, skin diseases, diabetes, cardiovascular diseases, stroke, age-related blindness, and lung disorders. Lungs are characterized by double vascularization and bronchial vasculature, associated with the thoracic aorta and the pulmonary system, which is part of the air/blood barrier of the lung tissue.

Vascular endothelial growth factor (VEGF) is one of the most potent angiogenesis-stimulating factors. VEGF interacts with three tyrosine kinase (TK) receptors, VEGFR–1, VEGFR–2, and VEGFR–3. VEGF, platelet-derived growth factor (PDGF), and fibroblast growth factor (FGF) have all been implicated in the pathogenesis of IPF [5]. Based on this, nintedanib is an inhibitor of PDGFRα and β, FGFR1–3, and VEGFR1–3, targets transforming growth factor-β (TGF-β), a pro-fibrotic cytokine that is involved in fibrotic tissue remodeling in asthma and other fibrotic diseases [6]. Despite using nintedanib, the prognosis of IPF is still not satisfactory. Currently, repeated lung injury or infection leads to aberrant wound healing causes massive extracellular matrix depositing and scarring of lung tissue eventually IPF. Correlation between VEGF and IPF aggravation is not well understood.

Under both hypoxic and hyperoxic conditions, the expression of VEGFR1 is increased in lung fibroblasts [7]. VEGF is also known to induce the mobilization of bone marrow (BM)-derived stem cells to injured tissue, where they induce angiogenesis [8, 9]. Furthermore, we demonstrated VEGFR1-TK signaling induced BM-derived VEGFR1+ cells induce compensatory lung growth and pulmonary fibrosis [10, 11]. In this review, we describe the involvement of VEGFR1-TK signaling in pulmonary fibrosis by using a bleomycin (BLM)-induced pulmonary fibrosis model and discussed the possibility of selective VEGFR1-TK inhibitor in future treatment.

## VEGF-A in pulmonary fibrosis

VEGF-A stimulates not only angiogenesis but also as lung development and homeostasis. VEGF-B, VEGF-C, VEGF-D, and placental growth factor (PlGF) are also thought to play a role in physiological lung development but have not been widely studied. VEGF-A binds two tyrosine kinase receptors VEGFR1 (Flt-1) and VEGFR2 (KDR) that regulate endothelial cell proliferation, migration, vascular permeability, secretion, and other endothelial functions. VEGF-A stimulates alveolar type II cell growth [12, 13], production of surfactant [14], and angiogenesis [15], and such a role for VEGF-A in lung repair following injury. On the other hand, VEGFR3 and its ligands VEGF-C and VEGF-D are recognized as a central molecular mechanism of lymphangiogenesis. TGF-β1-VEGF-C pathway in fibrosis is related in lymphangiogenesis in peritoneal mesothelial cells [16]. Cui et al. showed TGF-β1 suppresses VEGF-D expression in lung fibroblast that mediates remodeling in IPF [17]. VEGF-C expression is found to be significantly upregulated in response to TGF-β in renal tubule cells, peritoneal mesothelial cells, macrophages, and fibroblasts [18]. Although it is suggested that pro-lymphangiogenic factors including VEGF-C and VEGF-D are related to fibrosis, it remains to be clarified that the development of IPF is associated with lymphoangiogenesis. The overexpression of TGF-β1 induces rat peritoneal fibrosis, accompanied by neoangiogenesis, through the induction of VEGF-A production in mesothelial cells [19]. The development of peritoneal neoangiogenesis and fibrosis is mediated TGF-β1–VEGF-A pathway. Those reports indicate that the progression of IPF relates to angiogenesis.

**Fig. 1:**
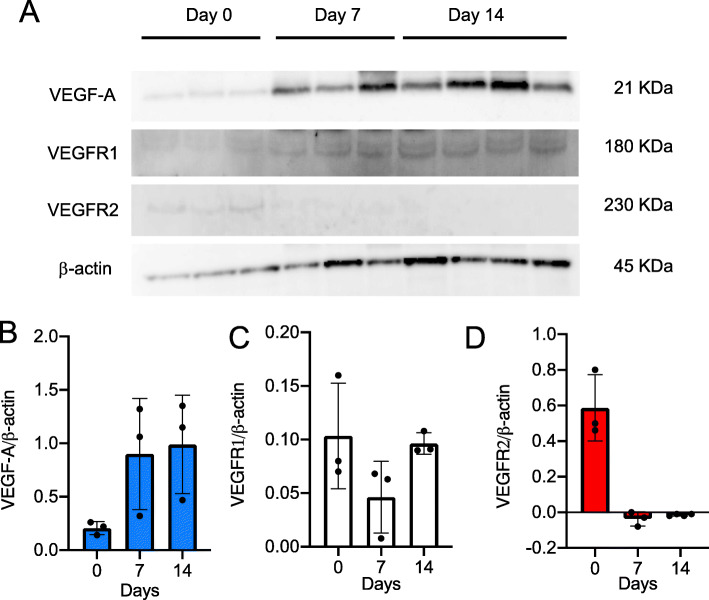
The protein level of VEGF receptors in the lung after BLM treatment. **a** The protein level of VEGF-A, VEGFR1, and VEGFR2 were evaluated by western blot analysis. **b**–**d** Statistical analysis of VEGF-A (**d**), VEGFR1 (**e**), and VEGFR2 (**f**) was determined and normalized to β-actin. Data are expressed as the mean ± SD (n = 3 mice per group) [11]

The relationship between VEGF-A expression and IPF remains to be explained. It appears to differ according to the compartment sampled. Several groups reported that reduced VEGF-A in the BALF of IPF patients compared to controls [20], whereas others have reported unchanged levels [21]. On the contrary, it is reported that VEGF-A levels in peripheral blood are associated with the severity and progression of IPF [22]. Enhanced expression of VEGF-A is correlated with increased alveolar capillary density in non-fibrotic regions of IPF lungs [23]. BLM-induced pulmonary fibrosis, commonly used to elucidate the mechanism of pulmonary fibrosis, demonstrates increase in VEGF-A and CD31 expression in the fibrotic regions [11]. Iyer et al. showed that anti-VEGF antibody (CBO-P11) significantly attenuates BLM-induced pulmonary fibrosis in vivo [24]. Anti-VEGF gene therapy, soluble flt-1 (sflt-1), specific receptors for VEGFR1, also attenuates BLM-induced pulmonary fibrosis [25]. However, it remains unknown about the effect of monoclonal antibodies for VEGF-A on pulmonary fibrosis in humans. Barratt et al. showed that VEGFR1 protein expression is enhanced in response to hypoxia but not in VEGFR2 [26]. These reports suggest that the expression of VEGFR1 is associated with pulmonary fibrosis. We found that the protein level of VEGFR1 gradually increased on day 14 but not in VEGFR2 (Fig. 1a–d). In addition, real-time PCR analysis showed that mRNA level of VEGFR1 expression was significantly increased on day 14. Base on this, we considered that the expression of VEGFR1 is related to fibrotic formation after BLM treatment.

## VEGFR1-TK signaling induces pulmonary fibrosis formation

VEGF-A binds to two receptors TK, VEGFR1 and VEGFR2. Both VEGFR1 deficient and VEGFR2 deficient mice die due to hematopoietic impairment on embryonic day (E) 8.5. VEGFR1-deficient embryo show overgrowth of endothelial cells and vascular disorganization and those cause death on E8.5 to 9.0. In contrast, knockout mice lacking the mice TK domain of VEGFR-1 (TKKO), lack only the signaling mediated by VEGFR1, show normal vascular development [27]. Base on this finding, it is a useful tool for using TKKO mice in research for understanding of pathological conditions especially downstream of VEGFR1-TK signaling. Considering previous results, we estimated the endogenous effect of VEGFR1-TK signaling on pulmonary fibrosis formation, using TKKO mice.

**Fig. 2:**
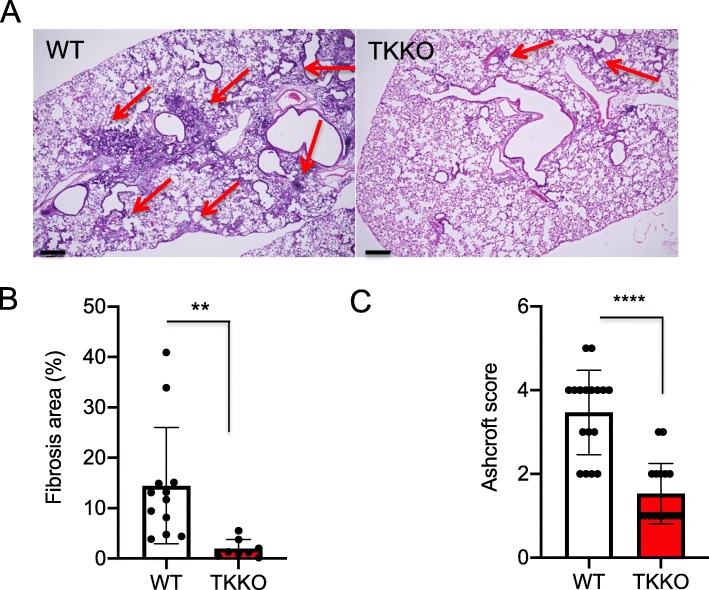
BLM-induced pulmonary fibrosis formation was suppressed in TKKO mice. **a** Hematoxylin-eosin staining of the lung in WT mice and TKKO mice on day 21 after BLM treatment. The red arrow indicates the fibrotic area. Bar = 200 μm. **b** The fibrotic area in the lung on day 21. Data are expressed as the mean ± SD (n = 8-12 mice per group). **p < 0.01 versus WT mice. **c** Ashcroft score WT mice and TKKO mice on day 21. Data are expressed as the mean ± SD (n = 14-17 mice per group). ****p < 0.0001 versus WT mice [11]

We showed that the percentage of fibrotic lung was significantly decreased in TKKO mice compared that in WT mice. In addition, we revealed that the Ashcroft score, the evaluation of pulmonary fibrosis, was significantly decreased in the TKKO mice (Fig. 2) [11].

**Fig. 3:**
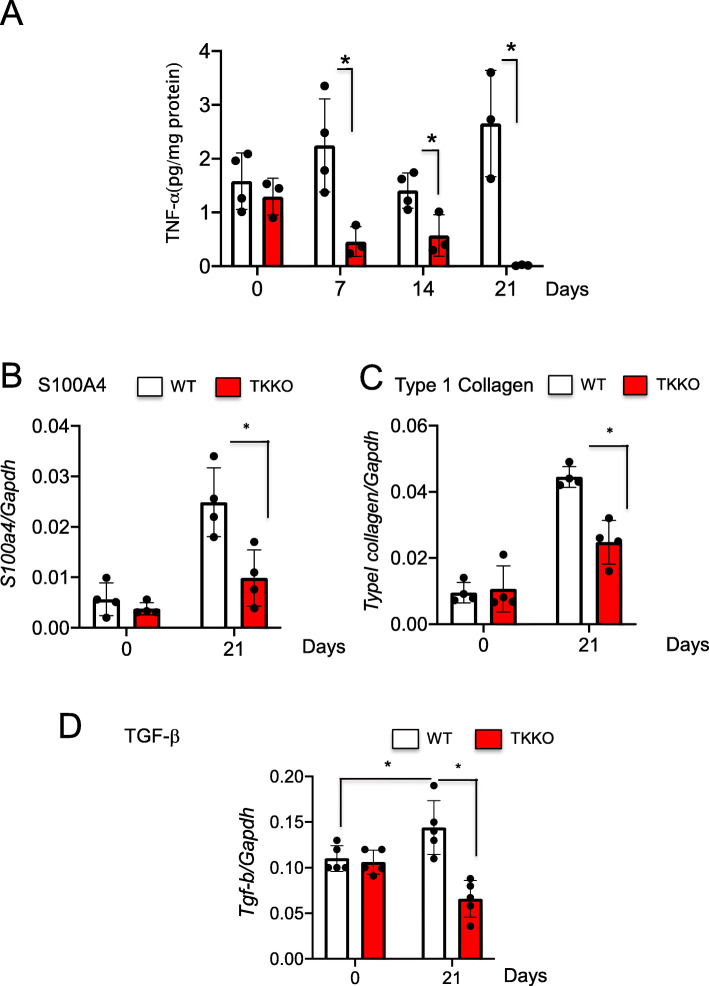
VEGFR1 TK signaling induces the expression of fibrosis-stimulating factors. **a** TNF-α levels in the lung after BLM treatment. Data are expressed as the mean ± SD (n = 4 mice per group) *p < 0.05 versus WT mice. **b**-**d** Expression of S100A4 (**b**), type I collagen (**c**), and TGF-β (**d**) in the lung on day 21 after BLM treatment. Data are expressed as the mean ± SD (n = 4-5 mice per group). *p < 0.05 versus WT mice [11]

Various growth factors and cytokines are involved in pulmonary fibrosis, including tumor necrosis factor alpha (TNF-α) and TGF-β [28]. TNF-α has both pro-inflammatory and fibrogenic properties and is highly expressed in IPF patients [29]. TNF-α upregulates TGF-β and collagen expression in both primary lung mesenchymal and epithelial cells. TNF-α produced by monocytes and macrophages are implicated in various pulmonary diseases, including pulmonary fibrosis [30]. We showed that the expression of several proinflammatory factors, TNF-α, and profibrotic factors, S100A4, type I collagen and TGF-β were significantly suppressed in TKKO mice on day 21 (Fig. 3) [11]. Those results suggested that VEGFR1-TK signaling is involved in pulmonary fibrosis.

Stromal cell-derived factor 1 (SDF-1), known as CXCL12, was first identified as a signal from the BM microenvironment to regulate hematopoiesis, including B lymphopoiesis [31]. SDF-1 is also known as an angiogenesis-stimulating factor. SDF-1 binds to specific receptors CXCR4 and CXCR7. CXCR7 is expressed in human endothelial cells including human umbilical vein endothelial cells (HUVECs) and human dermal microvascular endothelial cells (HMVECs). Zhang et al. showed that the SDF-1/CXCR7 axis induces angiogenesis by activating Akt signaling in HUVECs [32]. Increased levels of SDF-1 in the lung and plasma of patients with IPF correlate with numbers of circulating fibrocytes, which is previously also found in animals [33, 34]. The mobilization of circulatory fibrocytes and BM-derived primordial germ cells into injured lungs is controlled by the CXCL12/CXCR4 axis promoting the pathogenesis of pulmonary fibrosis [33, 35].

**Fig. 4:**
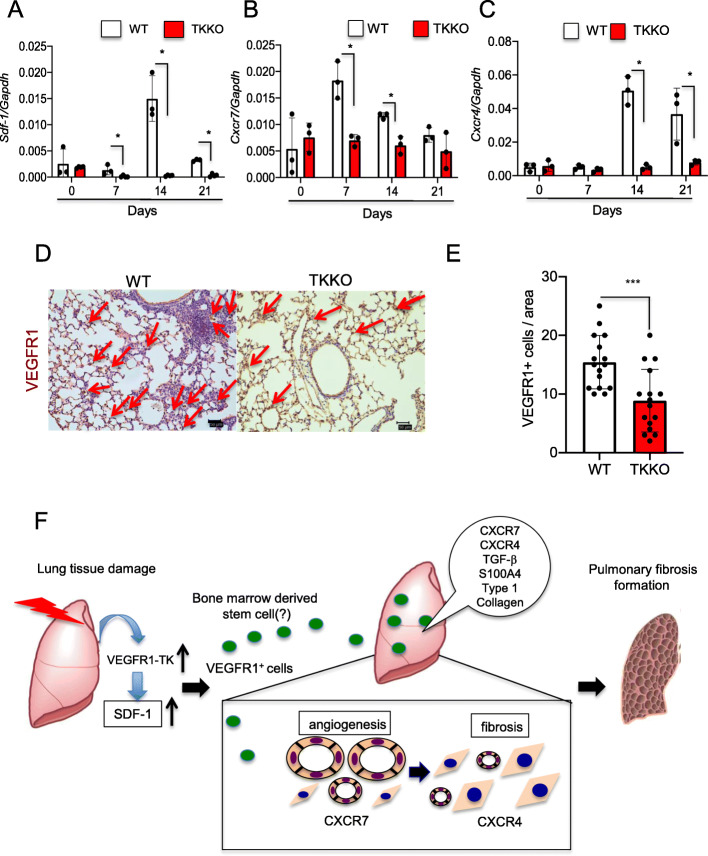
VEGFR1-TK signaling induces pulmonary fibrosis by VEGFR1^+^ cells accumulation through the expression of SDF-1/CXCR7/CXCR4 axis. The expression of SDF-1 (**a**), CXCR7 (**b**), and CXCR4 (**c**) in the lung on days 0, 7, 14, and 21 after BLM treatment. Data are expressed as the mean ± SD (n = 3 mice per group). *p < 0.05 versus WT mice. (**c**) Representative immunohistochemical staining of VEGFR1 in the lung in WT mice and TKKO mice on day 21 after BLM treatment. Bar = 50 μm (**d**). The number of VEGFR1^+^ cells in the lung WT mice and TKKO mice on day 21. Data are expressed as the mean ± SD (n = 16 mice per group). ***p < 0.001 versus WT mice. (**e**) Scheme of VEGFR1-TK signaling on pulmonary fibrosis formation after BLM treatment [11]

We demonstrated the pulmonary expression of SDF1 and CXCR7 was earlier than that of the CXCR4 expression, and that result suggested that CXCR7 is an early factor in the development of pulmonary fibrosis (Fig. 4a–c). And those expressions were suppressed in TKKO. The proregenerative CXCR7 pathway and the profibrotic CXCR4-pathway balance liver regeneration and fibrosis, supporting the hypothesis that inhibiting CXCR4 might ameliorate fibrogenesis [36]. Indeed, we showed treatment with CXCR4 antibody attenuates BLM-induced pulmonary fibrosis [11]. As with CD31, CXCR7 expresses in the pulmonary endothelial cells after BLM treatment. Further, activation of CXCR7 inhibits recruitment of VEGFR1^+^ macrophages, leading to the suppression of fibrosis during BLM-induced pulmonary fibrosis [36]. We demonstrated that the CXCR4 expression appeared late, and that result, i.e., expression of the CXCR7/CXCR4 axis, shows a causal relationship with pulmonary fibrosis development.

## VEGFR1-TK signaling accumulated VEGFR1^+^ cells

VEGF-A and SDF-1 are also known to induce the mobilization of VEGFR1^+^ cells from the BM. VEGFR1 is important for endothelial cell maintenance, vascular organization, and granuloma formation. VEGFR1 is required for the recruitment of hematopoietic precursors and migration of monocytes and macrophages. Endogenous progenitor cells are also essential to the organization of alveologenesis. In the lung tissue, VEGFR1 expression has been detected in alveolar type II cells from mice and rats, as well as from human fetuses [37, 38]. Activated myofibroblasts in lung fibrosis originate from resident fibroblasts and pericytes undergoing activation, alveolar epithelial cells under-going epithelial-to-mesenchymal transition, and circulating fibrocytes that are recruited from the BM. Cao et al. have shown that VEGFR1^+^ cells express CD11b and F4/80 recruit into the lung tissues after BLM treatment [39]. VEGFR1^+^ macrophages derived from BM are accumulated into ischemic tissues [9]. Regarding lung tissue, we showed that VEGFR1-TK signaling induces compensatory lung growth mediated by infiltrating BM-derived VEGFR1^+^ cells after pneumonectomy [10]. After BLM treatment, we demonstrated that VEGFR1^+^ cells are accumulated into the fibrotic regions of lung tissues (Fig. 4d, e).

AMD3100, a specific antagonist for CXCR4, directly inhibits the migration of human fibrocytes and suppressed pulmonary fibrosis formation treated with BLM [40]. Jin et al. reported that VEGFR1^+^ cells expressing CXCR4 are remobilized by hematopoietic stem cell factor through pro-MMP-9 activation [41]. We revealed that treatment of CXCR4 antibody reduced VEGFR1^+^ macrophages in the lung tissues in WT mice but not in TKKO mice [11]. This result showed that the attenuated pulmonary fibrosis formation in TKKO mice was dependent on the SDF-1/CXCR4 axis. The results of these experiments showed that VEGFR1-TK signaling induces pulmonary fibrosis by promoting the migration of VEGFR1^+^ cells, which is dependent on the SDF-1/CXCR4 axis (Fig. 4f).

## Tyrosine kinases inhibitor in clinical

Molecular-targeted therapy is designed to treat cancer by interrupting unique molecular abnormalities that derive cancer growth and metastasis. But recently, this treatment is given for other diseases such IPF. Molecular-targeted therapeutic agents are classified into small molecule drug and large molecule drug. Large molecule drugs, monoclonal antibodies, bind to the receptors localized on the cell surface. In contrast, small molecule drugs affect TKs, which play critical roles in diverse biological functions including adhesion, motility, proliferation, cell cycle controls, or cell death. Once something goes wrong with these functions cause cancer development, autoimmune diseases, and inflammatory diseases. Compared to monoclonal antibody, TK inhibitors may be useful for various diseases such as IPH and arthritis. In fact, TKs have been shown to play a critical role in the pathogenesis of IPF. Nintedanib is an inhibitor of PDGFRα and β, FGFR1–3, and VEGFR1–3. Nintedanib targets TGF-β, a profibrotic cytokine that is involved in fibrotic tissue remodeling in asthma, and other fibrotic diseases, and inhibits fibroblast proliferation induced by stimuli, such as PDGF, FGF, VEGF, and serum [6]. In clinical study, nintedanib showed a reduction in the rate of decline in lung function and a longer time to first exacerbation. The efficacy and safety of nintedanib in patients with IPF have been investigated in the phase II, randomized, dose-finding TOMORROW trial [42] and the two replicate, randomized, placebo-controlled, phase III INPULSIS trials [43, 44]. In patients with advanced IPF, compared with placebo, treatment with nintedanib is associated with less deterioration in health-related quality of life [45]. However, nintedanib was associated with high frequency of diarrhea, nausea, and vomiting. It is necessary to develop new drugs with less frequent side effect in the future.

## Conclusions

IPF is a lethal lung disease with poor prognosis. In spite of nintedanib is used in clinical, the treatment outcome does not meet expectation. We showed deletion of VEGFR1-TK signaling suppressed BLM-induced pulmonary fibrosis. These results demonstrated that the development of tyrosine kinase inhibitor that can specifically control VEGFR1-TK signaling must be an important issue and warrants further study. In future**,** selective VEGFR-1TK inhibitor may be a more useful drug to prevent and treat pulmonary fibrosis.

## Data Availability

Not applicable.

## Abbreviations

BLM: Bleomycin
BM: Bone marrow
CXCR4: Chemokine (C-X-C motif) receptor 4
CXCR7: C-X-C chemokine receptor type 7
VEGF: Vascular endothelial growth factor
VEGFR1: Vascular endothelial growth factor receptor 1
PDGF: Platelet-derived growth factor
SDF-1: Stromal cell-derived factor-1
TGF-β: Transforming growth factor-β
TK: Tyrosine kinase
